# Peritoneal spillage is not an issue in patients undergoing minimally invasive surgery for colorectal cancer

**DOI:** 10.1186/s12957-020-01882-z

**Published:** 2020-05-27

**Authors:** Peter C. Ambe, Joseph Kankam, Konstantinos Zarras

**Affiliations:** 1 Department of Visceral, Minimally Invasive and Oncologic Surgery, Marien Hospital Düsseldorf, Düsseldorf, Germany; 2 Department of Health, Faculty of Medicine, Witten/Herdecke University, Witten, Germany

**Keywords:** Colorectal cancer, Minimally invasive colorectal resection, Peritoneal tumor spillage, Tumor seeding, Peritoneal carcinomatosis

## Abstract

**Background:**

Surgery for colorectal cancer (CRC) is increasingly being performed via the minimally invasive route. However, reports of postoperative wound and port site seeding as well as peritoneal spillage have been worrisome. We investigated the risk of peritoneal spillage in patients undergoing laparoscopic surgery for CRC.

**Methods:**

Cytology specimens were gained from the retrieval bag following intracorporeal resection and specimen retrieval using an endoscopic retrieval bag. Histopathologic examination of the cytology specimens was performed for the presence of malignant cells.

**Results:**

Cytology specimens of 73 (34 female and 39 male) consecutive patients with a median age of 71 years were included for analysis. Advanced CRC in stages III and IV was present in 41% of the study population. Malignant cells were not found in any specimen.

**Conclusion:**

Laparoscopic oncologic resection of colorectal cancer is not a risk factor for peritoneal spillage. Minimally invasive oncologic colorectal resection is safe without the increased risk of peritoneal carcinomatosis.

## Introduction

Colorectal cancer (CRC) is one of the most common solid malignancies with about 70,000 new cases per year in Germany [1]. Unfortunately, an advanced disease with nodal (stage III) and distant (stage IV) metastases classified using the Union International Contre Le Cancer (UICC) classification is present in about 20% of cases at the time of diagnosis, rendering curative resection difficult [2]. Generally, the prognosis of CRC has been shown to be stage-dependent. Metastasis to the peritoneum among all sites of distant metastasis has been shown to be associated with an extremely poor prognosis with a median survival of just about 7 months [3]. Recurrently, modern oncologic strategies for the management of this subgroup encompassing cytoreductive surgery and intraoperative hyperthermic intraperitoneal chemotherapy (HIPEC) have evolved [4]. Data on this treatment option as well as experience from specialized oncologic centers are encouraging [5–7].

Oncologic resection with clear margins and systematic nodal dissection with complete mesocolic excision (CME) for colon cancer as described by Höhenberger et al. [8], and partial mesorectal excision (PME) for tumors of the rectosigmoidal junction and proximal rectum and total mesorectal excision (TME) as described by Heald et al. [9] for mid and low rectal cancer have been unequivocally proven to be determinants for recurrence-free survival (RFS), disease-free survival (DFS), and overall survival (OS). Traditionally, oncologic colorectal resection was openly performed. However, solid evidences from well-designed international double-blind multicenter studies like the CLASICC, COLOR, and COREAN trials have established the non-inferiority of laparoscopic oncologic colorectal resection in comparison to open surgery [10–12]. More so, the benefits of minimally invasive access in colorectal surgery in the era of enhanced recovery after surgery (ERAS) have contributed to increased acceptance of the minimally invasive approach [13]. Currently, the minimally invasive approach has evolved to be the standard for the management of patients with CRC in specialized centers.

Despite the abovementioned advantages, the initial acceptance of minimally invasive oncologic colorectal resection was low. Reported cases of port site metastasis in the abdominal wall questioned the safety of the technique [14]. Besides, reports of rare cases of peritoneal spillage and port site seeding following laparoscopic resection of CRC have been worrisome [15, 16]. Theoretically, peritoneal spillage and port site seeding may occur as a result of pressure on the tumor during dissection and/or retrieval of the colectomy specimen. This disastrous complication may be secondary to surgery and therefore may be coined as a perioperative complication. Both peritoneal spillage and port site seeding dampened many proponents of minimally invasive management of CRC. The aim of this study was to investigate the risk of peritoneal tumor spillage during laparoscopic oncologic resection for CRC.

## Methods

Minimally invasive oncologic resection is the standard procedure for patients with CRC in our department. According to our departmental standards, all patients diagnosed with CRC are presented at the multidisciplinary oncologic board prior to treatment. Since this constituted our standard clinical practice, ethics approval was waived.

All consecutive cases of CRC undergoing surgical resection via minimally invasive access within the period of observation from March to October 2018 were included in this study. Cases with open surgery and all cases converted from laparoscopic to open surgery were excluded from analysis. Cases with suspected peritoneal carcinomatosis during surgery and cases undergoing palliative procedures were also excluded. All cases were prospectively recorded in a database.

Oncologic resection consisted of CME for colon cancer, PME for cancers of the rectosigmoidal junction and proximal rectum, and TME for mid and low rectal cancer. Intracorporeal resection of the involved segment was performed after radical dissection using laparoscopic lineal stapling devices. The specimen was removed from the abdominal cavity using an endoscopic retrieval bag (endobag). The colectomy or rectum specimen was taken out of the endobag for inspection. Hereafter, 20 ml normal saline was instilled into the endobag, which was then reclosed and gently shaken for 30 s. The resulting content was collected in a cytology vessel and sent for histopathology.

Post-surgical oncologic management with regard to adjuvant chemotherapy and follow-up was in accordance with international standards.

## Results

Ninety-eight cases with CRC were managed in our department during the period of investigation. Palliation and local ablative procedures were performed in seven cases.

**Fig. 1:**
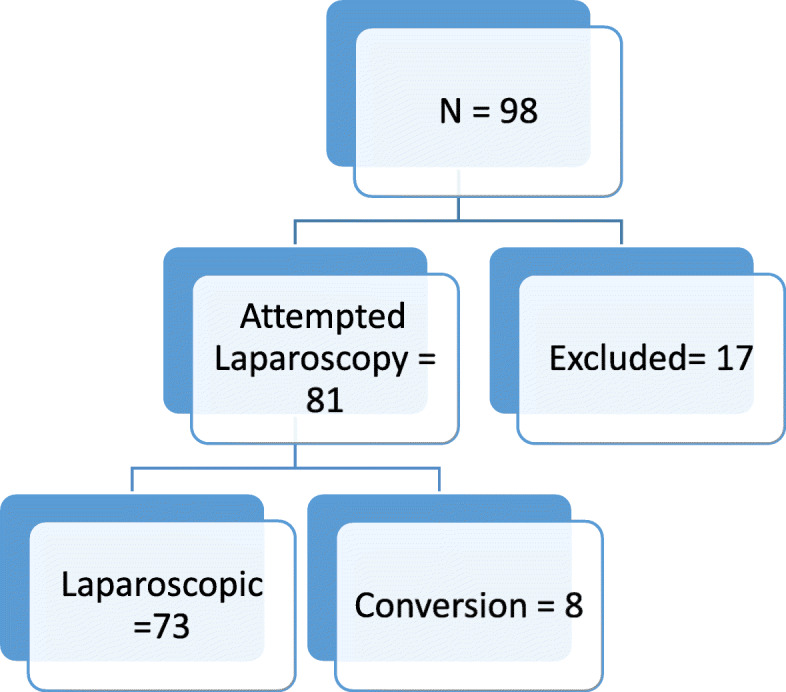
Distribution of the study population. Reasons for exclusion: palliation surgery 4×, transanal resection 2×, definitive radiation 1×, and primary open surgery 10×

Attempted laparoscopic resection was performed in 81 cases. Conversion to open surgery was performed in eight patients, putting the rate of conversion at 9.8%. Primary open surgery was performed in 10 cases. The distribution of the study population is presented in Fig. 1.

The study group consisted of 73 (34 female and 39 male) patients undergoing laparoscopic oncologic resection for CRC. The median age at the time of surgery was 71 years (range 38–90 years). A summary of the postoperative histopathologic findings of the study population is presented in Table 1. Cytology was negative in all cases. No case of peritoneal recurrence has been recorded in the study population within a follow-up period of 14–20 months.

**Table 1:** Clinicopathologic findings of the study population. M1: 10× hepatic metastasis, 1× metastasis to the ovary, 1× metastasis in the large omentum, and 1× metastasis in a distant lymph node

Features	Number of cases (%)
Tumor location	
Right colon	26 (35.7)
Left colon	22 (30.1)
Rectum	25 (34.2)
AJCC stage	
T1/T2	20 (27.4)
T3	46 (63.0)
T4	07 (9.6)
Nodal status	
N0	46 (63.0)
N1	14 (19.2)
N2	13 (17.8)
Distant metastasis	
M0	60 (82.2)
M1	13 (17.8)

## Discussion

Oncologic colorectal resection via the minimally invasive route is increasingly being employed for the management of patients with CRC. Reports of peritoneal spillage and port site metastasis secondary to laparoscopic management of CRC have been a cause of concern. We investigated the risk of peritoneal seeding in patients undergoing laparoscopic oncologic resections for CRC. No case of spillage was found in this series irrespective of the tumor stage.

The current literature on peritoneal spillage and port site seeding following laparoscopic resection of CRC is limited to case reports [17–19]. Nonetheless, these devastating cases dampened the initial enthusiasm of laparoscopic oncologic colorectal surgery and questioned the safety of minimally invasive surgery for oncologic entities including CRC [20]. Besides published case reports, data on peritoneal spillage and port site seeding is limited.

The largest study looking into wound, port site, and peritoneal recurrence is a meta-analysis from 2013 by Zanghi et al. comparing the outcomes of 2431 laparoscopic and 2176 open cases. No differences were found between the laparoscopic (27/2431, 0.01%) and the open (17/2176, 0.01%) groups with regard to wound, port site, and peritoneal recurrence [15]. The main finding from this meta-analysis argues for the safety of laparoscopic resection of CRC.

No study so far has purposely investigated the risk of peritoneal spillage in patients undergoing laparoscopic oncologic colorectal resection. This study therefore, to the best of our knowledge, represents the first prospectively designed study to investigate the risk of peritoneal spillage in patients undergoing laparoscopic resection for CRC.

Cytology specimens of 73 consecutive patients undergoing laparoscopic oncologic resection for CRC were investigated in this study. All cytology specimens were negative for malignant cells. The risk of peritoneal spillage has been thought to be associated with the tumor size. The majority of patients with peritoneal recurrence following laparoscopic resection of CRC reported so far underwent surgery for advanced CRC, mostly stage C according to Dukes’ classification [21–23]. In most cases, peritoneal recurrence was diagnosed within 12 months. This tendency could not be confirmed in our study. Postoperative histopathology confirmed stage III and IV tumors in 41% of our study population. Even in these cases, cytology specimens were negative for malignant cells. So far, no case of peritoneal recurrence has been seen within the follow-up period of more than 14 months. This intriguing finding must be interpreted as an argument for the safety of minimally invasive surgery even in selected patients with large tumors.

Recently published data suggests some advantages of intracorporeal over extracorporeal ileocolic anastomosis following laparoscopic right colectomy with respect to anastomotic leakage, early return of bowel movement, and length of incision [24–26]. Thus, the trend is towards total intracorporeal resection and anastomosis. The main finding from our study is strongly in accordance with the development in minimally invasive oncologic colorectal surgery.

The lack of malignant cells in all cytology specimens documented in our study has prompted us to change our departmental standards with regard to colectomy specimen retrieval. Endoscopic retrieval bags (endobags) are no longer routinely used in our department for specimen retrieval. This change of concept has reduced our departmental expenditure for endobags.

The time interval between cytology sample collection and histopathologic analysis is not documented in this series. This may be a possible limitation due to the fact that cells may disintegrate while being stored in saline. False negative cytology therefore may have occurred due to cell disintegration in some cases. The duration of a rather short follow-up (14 months) constitutes another limitation in this study. We therefore would be reporting on the long-term survival data of this collective in the future.

Taken together, cytology of patients undergoing laparoscopic surgery for colorectal cancer in this study was negative in all cases. This finding suggests that laparoscopic resection of CRC may not increase the risk of peritoneal spillage as long as oncologic standards are respected.

## Conclusion

Laparoscopic oncologic resection of colorectal surgery is not a risk factor for peritoneal spillage. Minimally invasive oncologic colorectal resection is safe without increased risk of peritoneal carcinomatosis.

## Data Availability

The dataset supporting the conclusions of this article is included within the article.

## Abbreviations

AJCC: American Joint Committee on Cancer
CME: Complete mesocolic excision
CRC: Colorectal cancer
DFS: Disease-free survival
ERAS: Enhanced recovery after surgery
PME: Partial mesorectal excision
HIPEC: Hyperthermic intraperitoneal chemotherapy
OS: Overall survival
RFS: Recurrence-free survival
TME: Total mesorectal excision
UICC: Union International Contre Le Cancer

## References

[1] Ambe PC, Jansen S, Zirngibl H (2017). New trend in colorectal cancer in Germany: are young patients at increased risk for advanced colorectal cancer?. World J Surg Oncol.

[2] Stewart B, Wild CP (2015). World cancer report 2014. World.

[3] Jayne DG, Fook S, Loi C, Seow-Choen F (2002). Peritoneal carcinomatosis from colorectal cancer. Br J Surg.

[4] van Eden WJ, Kok NFM, Woensdregt K, Huitema ADR, Boot H, Aalbers AGJ (2018). Safety of intraperitoneal Mitomycin C versus intraperitoneal oxaliplatin in patients with peritoneal carcinomatosis of colorectal cancer undergoing cytoreductive surgery and HIPEC. Eur J Surg Oncol.

[5] Elias D, Blot F, El Otmany A, Antoun S, Lasser P, Boige V (2001). Curative treatment of peritoneal carcinomatosis arising from colorectal cancer by complete resection and intraperitoneal chemotherapy. Cancer.

[6] Esquivel J, Sticca R, Sugarbaker P, Levine E, Yan T, Alexander R (2007). Cytoreductive surgery and hyperthermic intraperitoneal chemotherapy in the management of peritoneal surface malignancies of colonic origin: a consensus statement. Ann Surg Oncol.

[7] Piso P, Bektas H, Werner U, Schlitt HJ, Kubicka S, Bornscheuer A (2001). Improved prognosis following peritonectomy procedures and hyperthermic intraperitoneal chemotherapy for peritoneal carcinomatosis from appendiceal carcinoma. Eur J Surg Oncol.

[8] Hohenberger W, Weber K, Matzel K, Papadopoulos T, Merkel S (2009). Standardized surgery for colonic cancer: complete mesocolic excision and central ligation--technical notes and outcome. Color Dis.

[9] Heald RJ, Moran BJ, Ryall RD, Sexton R, MacFarlane JK (1998). Rectal cancer: the Basingstoke experience of total mesorectal excision, 1978-1997. Arch Surg.

[10] Jeong SY, Park JW, Nam BH, Kim S, Kang SB, Lim SB (2014). Open versus laparoscopic surgery for mid-rectal or low-rectal cancer after neoadjuvant chemoradiotherapy (COREAN trial): survival outcomes of an open-label, non-inferiority, randomised controlled trial. Lancet Oncol.

[11] Jayne DG, Guillou PJ, Thorpe H, Quirke P, Copeland J, Smith AM (2007). Randomized trial of laparoscopic-assisted resection of colorectal carcinoma: 3-year results of the UK MRC CLASICC trial group. J Clin Oncol.

[12] Hazebroek EJ (2002). Color. Surg Endosc Other Interv Tech.

[13] King P, Blazeby J, Ewings P, Franks P, Longman R, Kendrick A (2006). Randomized clinical trial comparing laparoscopic and open surgery for colorectal cancer within an enhanced recovery programme. Surgery S.

[14] Paolucci V, Schaeff B, Schneider M, Gutt C (1999). Tumor seeding following laparoscopy: international survey. World J Surg.

[15] Zanghi A, Cavallaro A, Piccolo G, Fisichella R, Di Vita M, Sparta D (2013). Dissemination metastasis after laparoscopic colorectal surgery versus conventional open surgery for colorectal cancer: a metanalysis. Eur Rev Med Pharmacol Sci.

[16] Ouchi A, Komori K, Kimura K, Kinoshita T, Ito S, Abe T (2017). Solitary distant peritoneal metastasis of cecal cancer after laparoscopic colectomy: a case report. J Med Investig.

[17] Jacquet P, Averbach AM, Jacquet N (1995). Abdominal wall metastasis and peritoneal carcinomatosis after laparoscopic-assisted colectomy for colon cancer. Eur J Surg Oncol.

[18] Jacquet P, Averbach AM, Stephens AD, Sugarbaker PH (1995). Cancer recurrence following laparoscopic colectomy. Report of two patients treated with heated intraperitoneal chemotherapy. Dis Colon Rectum.

[19] Walsh DC, Wattchow DA, Wilson TG (1993). Subcutaneous metastases after laparoscopic resection of malignancy. Aust N Z J Surg.

[20] Bergamaschi R, Myrvold HE (1997). Laparoscopic surgery for cure of colorectal cancer. Surg Endosc.

[21] Guillou PJ, Darzi A, Monson JR (1993). Experience with laparoscopic colorectal surgery for malignant disease. Surg Oncol.

[22] Nduka CC, Monson JR, Menzies-Gow N, Darzi A (1994). Abdominal wall metastases following laparoscopy. Br J Surg.

[23] Ramos JM, Gupta S, Anthone GJ, Ortega AE, Simons AJ, Beart RW (1994). Laparoscopy and colon cancer. Is the port site at risk? A preliminary report. Arch Surg.

[24] Allaix ME, Degiuli M, Bonino MA, Arezzo A, Mistrangelo M, Passera R (2019). Intracorporeal or extracorporeal ileocolic anastomosis after laparoscopic right colectomy: a double-blinded randomized controlled trial. Ann Surg.

[25] Milone M, Elmore U, Vignali A, Gennarelli N, Manigrasso M, Burati M (2018). Recovery after intracorporeal anastomosis in laparoscopic right hemicolectomy: a systematic review and meta-analysis. Langenbeck's Arch Surg.

[26] Vignali A, Elmore U, Lemma M, Guarnieri G, Radaelli G, Rosati R (2018). Intracorporeal versus extracorporeal anastomoses following laparoscopic right colectomy in obese patients: a case-matched study. Dig Surg.

